# Collision tumor of thyroid: A case report of synchronous papillary and follicular thyroid carcinoma

**DOI:** 10.1097/MD.0000000000043982

**Published:** 2025-08-22

**Authors:** Dabin Kim, Susie Chin, Cheol-Wan Lim, Zisun Kim, Sung-Mo Hur

**Affiliations:** a Department of Surgery, Soonchunhyang University Bucheon Hospital, Soonchunhyang University College of Medicine, Bucheon, Gyeonggi-do, Korea; b Department of Pathology, Soonchunhyang University Bucheon Hospital, Soonchunhyang University College of Medicine, Bucheon, Gyeonggi-do, Korea.

**Keywords:** collision tumor, follicular thyroid carcinoma, papillary thyroid carcinoma, thyroid neoplasms

## Abstract

**Rationale:**

Collision tumors involving differentiated thyroid carcinomas are exceedingly rare, despite the overall high prevalence of thyroid cancer.

**Patient concerns:**

A 65-year-old man was incidentally diagnosed with thyroid cancer during a staging workup for gastric cancer.

**Diagnoses:**

The histopathologic examination revealed 2 coincident cancers of the left thyroid lobe: papillary carcinoma (pT1bN1a) and follicular carcinoma (pT2N0).

**Interventions:**

The patient underwent total thyroidectomy with central lymph node dissection, followed by thyroid hormone suppression and radioactive iodine therapy.

**Outcomes:**

No postoperative complications or cancer recurrence has been observed to date.

**Lessons:**

This case highlights the diagnostic challenges and surgical considerations associated with collision tumors of the thyroid. Appropriate management remains controversial; therefore, further case studies and consensus guidelines are necessary.

## 1. Introduction

Thyroid cancer (TC) is the most common endocrine cancer worldwide.^[1]^ Well-differentiated TCs, comprising papillary thyroid cancer (PTC) and follicular thyroid cancer (FTC), account for approximately 90% of all TCs and are typically known to have an indolent clinical course. In contrast, medullary thyroid cancer (MTC), poorly differentiated thyroid cancer, and anaplastic thyroid cancer represent <10% of TCs and are associated with aggressive behavior.^[2]^ Collision tumors are rare pathological conditions that coexist with histologically discrete tumors in a single organ. Collision tumors have been documented in various organs throughout the body, including kidneys, stomach, skin, liver, and ovaries.^[3]^ Likewise, collision tumors are rare in the thyroid gland, despite the relatively high incidence of TC. Here, we report the case of a male patient with an incidentally discovered collision tumor of PTC and FTC. Additionally, a brief literature review was conducted on cases of thyroid collision tumors involving papillary and follicular carcinomas.

## 2. Case presentation

**Figure 1.**
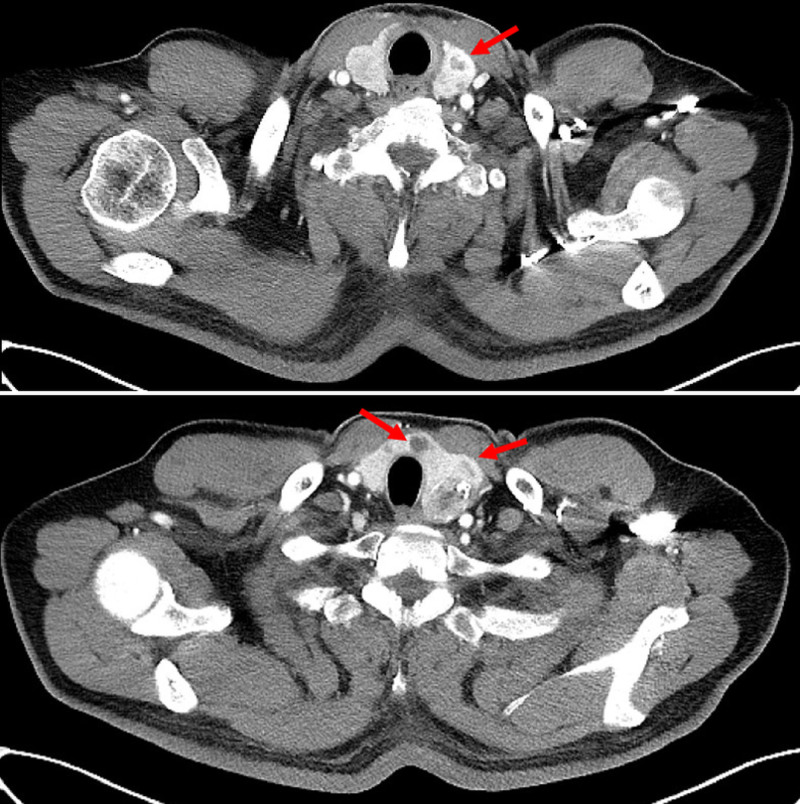
Multiple thyroid nodules were found in chest CT during staging workup of gastric cancer.

**Figure 2.**
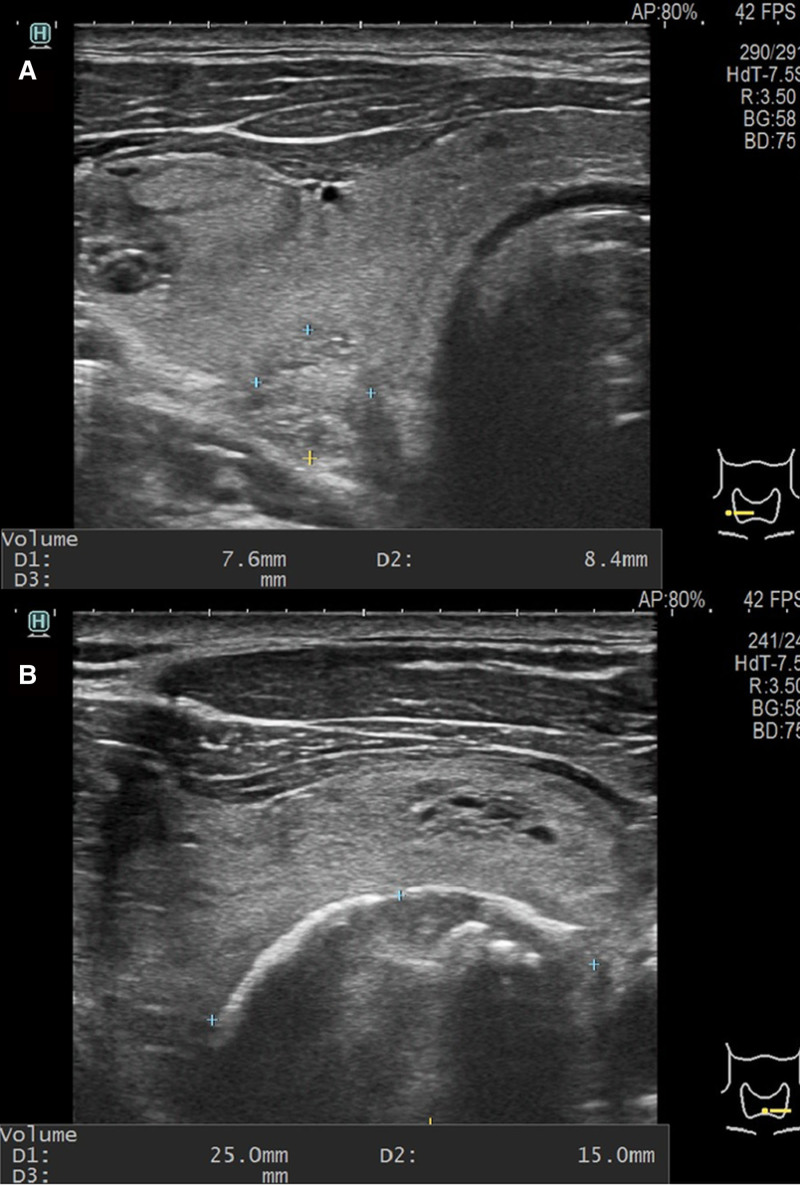
(A) An approximately 8 mm nodule was found in the right lower pole (TIRADS category 5). (B) A 25 mm nodule of the left lower pole (TIRADS category 4). TIRADS = thyroid imaging reporting and data system.

**Figure 3.**
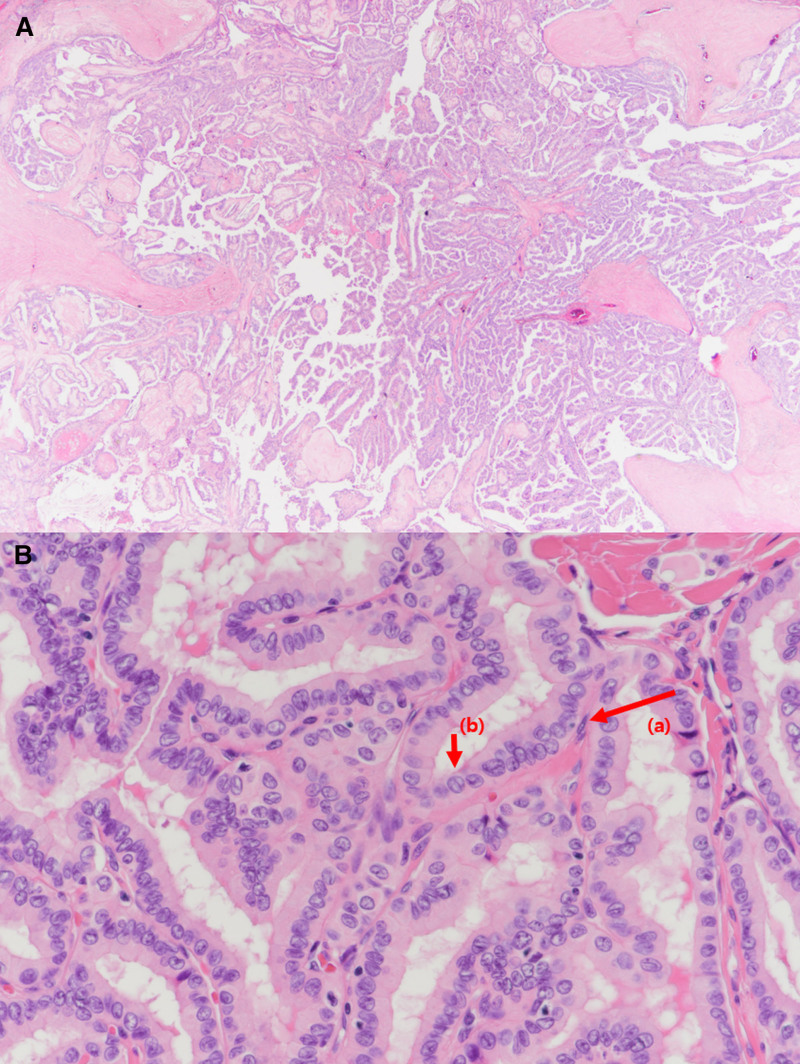
(A) Low (H&E, ×40) and (B) high (H&E, ×200) magnification of papillary carcinoma. Multiple papillae are noted, composed of fibrovascular stalk (A) lined by tumor cells. The tumor cells show typical nuclear grooves (B) and irregular nuclear membranes.

**Figure 4.**
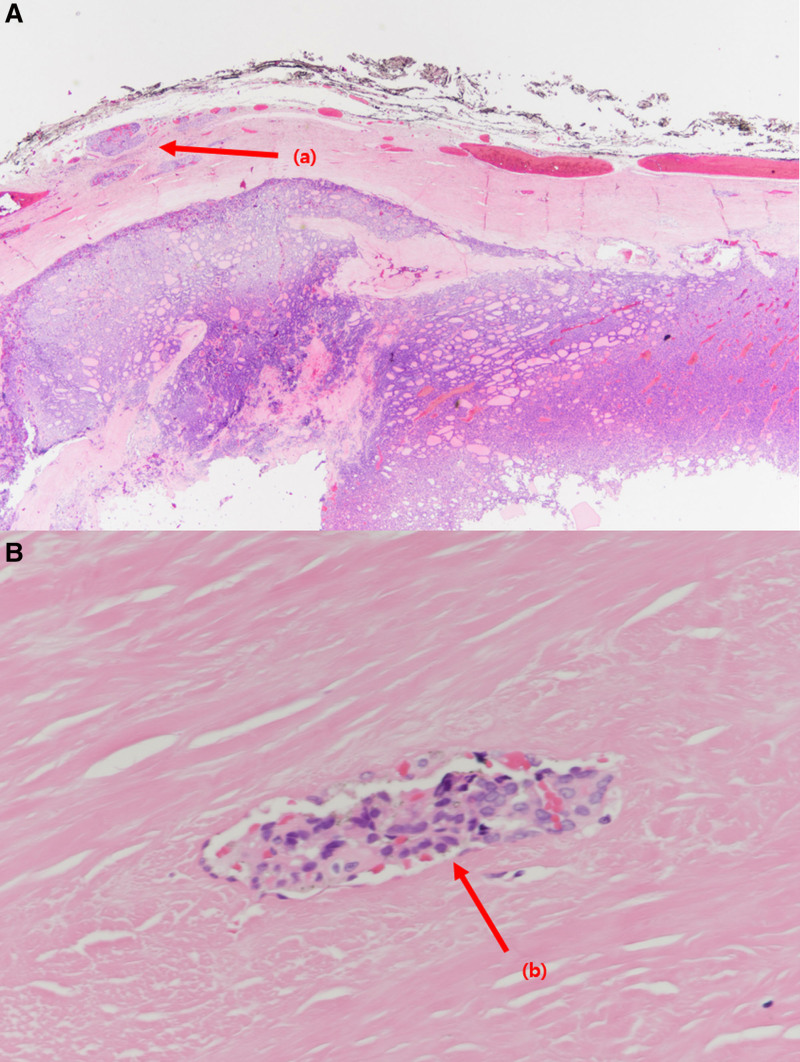
(A) Low (H&E, ×20) and (B) high (H&E, ×200) magnification of follicular carcinoma. The tumor composed of microfollicles, and tumor cell nests (A) are found in the extracapsular area. The tumor cell cluster (B) is attached to the vessel wall, lined by endothelial cells.

**Figure 5.**
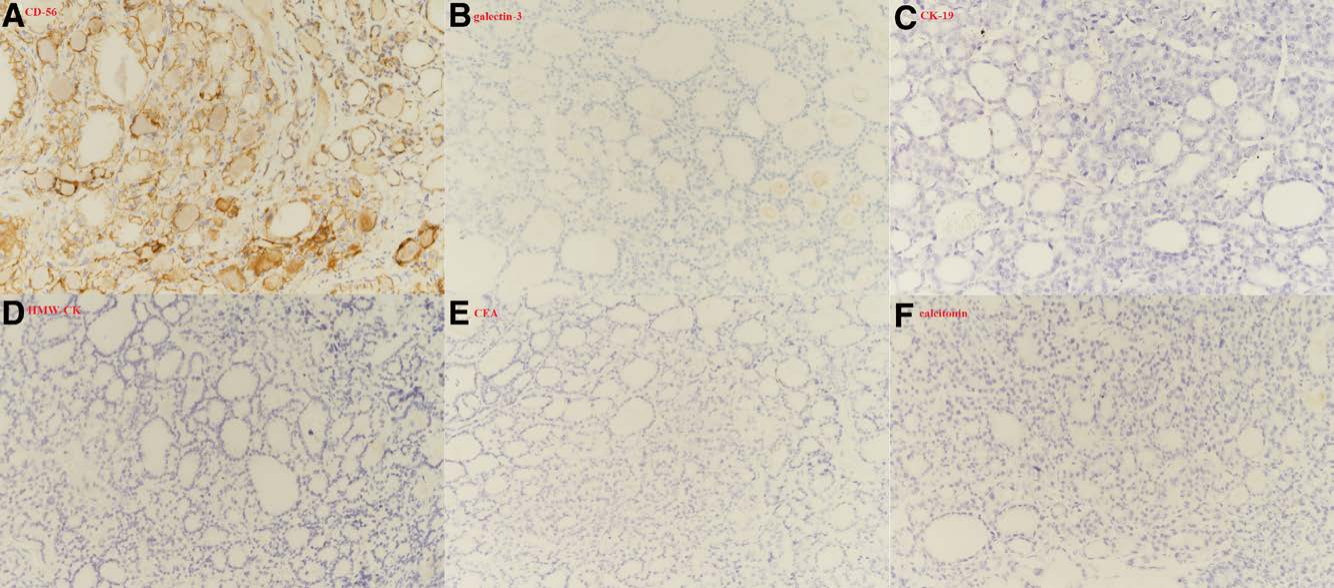
Immunohistochemical staining (×200) of the follicular carcinoma showed positivity for (A) CD-56, and negativity for (B) galectin-3, (C) CK-19, (D) HMW-CK, (E) CEA, and (F) calcitonin. CEA = carcinoembryonic antigen, HMW-CK = high-molecular-weight cytokeratin.

A 65-year-old man was referred to the endocrine surgery department for the incidental detection of TC during a staging workup for gastric cancer. The patient had a medical history of hypertension and diabetes mellitus type 2, and did not have any family history of TC or radiation to the neck area. No abnormal findings were observed upon physical examination. Multiple thyroid nodules were detected on chest computed tomography (Fig. 1), and the patient underwent thyroid ultrasonography. Approximately 9 thyroid nodules were found, 2 were categorized as 4 and 5 according to the thyroid imaging reporting and data system by the American College of Radiology, and the others were categorized as 3. Two nodules classified as thyroid imaging reporting and data system categories 4 and 5 were subjected to fine-needle aspiration and BRAF mutation testing. The first nodule (A) of category 5 was located in the right lower pole, 7.6 × 8.4 × 7.6 mm in size, and had a speculated margin and microcalcification. The second category 4 nodule (B), located in the left lower pole, was 25 × 15 × 20 mm in size, hypoechoic, and macrocalcified (Fig. 2). Nodule (A) was revealed as a follicular lesion of undetermined significance with cytological atypia (Bethesda category III), and nodule (B) was suspected to be a papillary carcinoma (Bethesda category V). BRAF mutation analysis using PNA-mediated real-time PCR of fine-needle aspiration specimens revealed no detectable mutations in either nodule. No distant or suspicious lymph node metastases were observed. Laboratory tests revealed low thyroid-stimulating hormone levels of 0.1 µIU/mL (reference range: 0.39–5.44), anti-thyroglobulin antibody levels of 12.6 U/mL (reference range: 0–80), and anti-microsomal antibody levels of <9.0 U/mL (reference range: 0–50). Because suspicious nodules were located in the bilateral thyroid lobes, the patient underwent total thyroidectomy with central lymph node dissection, combined with proximal gastrectomy for concomitant gastric cancer. The final histopathologic result revealed 2 coincident cancers of the left thyroid, a 1.2-cm sized papillary carcinoma with extrathyroidal extension (Fig. 3) and a 2.5-cm sized follicular carcinoma with capsular and lymphovascular invasion, which correlated with the nodule (B) (Fig. 4). Two of the 10 central lymph nodes were diagnosed with metastatic papillary carcinoma. The pathological stage was pT1bN1a for PTC, and pT2N0 for FTC according to the TNM staging system (American Joint Committee on Cancer, 8th edition). Immunohistochemical staining of the FTC was positive for CD-56 and negative for galectin-3, CK-19, high-molecular-weight cytokeratin, carcinoembryonic antigen, and calcitonin (Fig. 5). Nodule (A) classified as Bethesda category III was subsequently diagnosed as nodular hyperplasia. Thyroid suppression treatment was initiated with 0.15 mg of levothyroxine on postoperative day 3, and the thyroid-stimulating hormone level was successfully suppressed to <0.1 µIU/mL. The patient recovered without any postoperative complications. Radioactive iodine treatment with high-dose I-131 (100 mCi) was administered because of lymph node metastasis and extrathyroidal extension. After 24 months of follow-up, the thyroglobulin and anti-thyroglobulin levels were 0.13 ng/mL (reference range: 0–35) and 12.0 U/mL (reference range: 0–80), respectively. No postoperative complication or cancer recurrence was observed.

## 3. Discussion

TC is the most common endocrine cancer worldwide.^[1]^ The majority of TCs originate from the follicular epithelium of thyroid and include PTC, FTC, and anaplastic thyroid cancers. MTC arises from parafollicular or C cells of thyroid gland.^[2]^ The most common TCs are differentiated TC, comprising PTC and FTC. The incidence of PTC is increasing worldwide, accounting for approximately 90% of all cases, with FTC accounting for <5%.^[4]^ Differentiated TC has a well-documented indolent clinical course, with a relatively good prognosis; however, each type has a different spreading pattern—PTC tends to spread through the lymphatic system, and FTC tends to spread hematogenously.^[5]^

Some cases have reported the coexistence of different types of cancer in a single organ, classified into collision, mixed, and composite tumors based on the singularity and histologic separation of the tumor.^[6]^ Collision tumors are those that coexist in a single organ with histologically distinct and morphologically independent neoplasms.^[7]^ Collision tumors can occur in various organs of the body. However, collision tumors of the thyroid are rare, accounting for <1% of thyroid malignancies. Lamberg et al first reported a thyroid collision tumor in 1981, comprising both MTC and PTC.^[8,9]^ Since the first reported case, most thyroid collision tumors have been a combination of MTC and PTC. Notably, Ryan et al reported 20 cases of MTC with PTC among 33 cases of thyroid collision tumors as of 2015.^[10]^

Several hypotheses have been proposed to explain the development of thyroid collision tumors, including the predisposing theory, the stem cell theory, the random collision theory, and the hostage theory (neoplastic coercion).^[3,6,11]^ Given the pivotal role of genetic alterations in TC, some studies have suggested a potential relationship between RET proto-oncogenes and collision tumors of MTC and PTC.^[12]^ However, none of these theories fully account for the phenomenon, and a comprehensive understanding may require an integrative approach combining elements from multiple theories.

Collision tumors involving PTC and FTC are relatively rare among thyroid collision tumors, with only 16 case reports documented to date, similar to our case.^[3,5,11,13–25]^ Table 1 shows the specific characteristics of patients with PTC and FTC collision tumors among the major case reports. Distant and nodal metastases have also been reported. Notably, only 1 case of nodal metastasis of both PTC and FTC, and 2 cases of distant metastasis of PTC did not coincide with the typical spread pattern of the disease. Three patients experienced recurrence or distant metastases during follow-up. However, information regarding immunohistochemical staining and genetic alterations is relatively lacking.

**Table 1:** Previously reported cases of thyroid collision tumor of PTC and FTC.

Reference	Sex	Age	Clinical presentation	FNA (Bethesda category)	PTC-pathology	FTC-pathology	Spread	Other thyroidal pathologic findings	TFT	Treatment	RAI therapy	Thyroid function suppression	Prognosis
					PTC-IHC/genetic study	FTC-IHC/genetic study							
Plauche et al^[5]^	F	62	- Palpable neck nodule - Palpitation - Sweating	IV	Left lobe pT1mN0	Left lobe/right lobe pT3aN0, vascular invasion (+), capsular invasion (+)	(‐)	Hashimoto thyroiditis	WNL	LL → completion TT	(+)	N/A	No recurrence
					(‐)	(‐)							
Thomas et al^[3]^	M	33	Anterior neck swelling	IV	Right lobe pT1aN0	Right lobe pT3aN0, capsular invasion (+)	(‐)	Hashimoto thyroiditis	N/A	RL → completion TT	(-)	(+)	↑Uptake in RAI scan at thyroid bed, →RAI ablation done
					(‐)	(‐)							
Cracolici et al^[15]^	F	63	Flank pain	(‐)	Left lobe pT1bN1a	Right lobe pT1bN0	Rib/T11 metastasis (FTC), lymph node metastasis (PTC)	(‐)	WNL	TT + CND	(+)	N/A	Metastasis to liver, sacrum, T12
					BRAF (+)	NRAS (+)							
Feng et al^[13]^	F	40	Incidental finding in health checkup	(‐)	Right lobe pT1bN0	Left lobe	(‐)	(‐)	WNL	TT + CND	(‐)	(+)	No recurrence
					(‐)	CK-19, thyroglobulin (+), Ki-67 1%							
He et al^[18]^	M	71	Incidental adrenal mass on CT	III → II	Left lobe pT1aN0	Right lobe pT3aN0, lymphatic invasion (+), ETE (‐)	Adrenal metastasis (FTC)	(‐)	N/A	TT	(+)	(+)	No recurrence
Vlaenderen et al^[17]^	F	12	Painless neck swelling	(‐)	Left lobe pT2N0, capsular invasion (+), vascular invasion (+)	Left lobe	(‐)	(‐)	↑fT4, ↓TSH	LL → completion TT	(‐)	(‐)	No recurrence
					(‐)	HBME-1 (+)							
Carrion et al^[20]^	M	56	Right shoulder pain	V	Right lobe pT2N0	Left lobe pT1bN0	Bone metastasis (PTC)	(‐)	N/A	TT + neck dissection	(+)	(+)	No recurrence
Fonseca et al^[21]^	F	71	Pain and swelling over the left temporal region	(‐)	Left lobe pT1aN0	Left lobe pT1bN0, capsular invasion (+)	Soft tissue metastasis—left temporal (PTC)	(‐)	N/A	TT	(+)	N/A	No recurrence
					BRAF (+)	(‐)							
Stenman et al^[22]^	F	43	Right neck swelling	VI	Right lobe pT2mN1b, ETE (‐), LVI (‐)	Left lobe pT1bN1b, capsular invasion (+)	Lymph node metastasis (PTC/FTC)	(‐)	N/A	TT + neck dissection	(+)	(+)	No recurrence
					BRAF (+), Ki-67 5.6%	NRAS (+), Ki-67 3%							
Ahuja et al^[23]^	F	35	- Scalp swelling - Anterior neck swelling	V	Left lobe pT2N1	Entire gland pT4N0	Bone metastasis (FTC), lymph node metastasis (PTC)	(‐)	WNL	TT + CND	(+)	(+)	No recurrence
					(‐)	(‐)							
Kawasaki et al^[24]^	F	69	- Dysphagia - Right cervical mass	V	Right lobe pT4aN1b	Left lobe pT3aN0, capsular invasion (+)	Lymph node metastasis (20/32) (PTC)		N/A	TT + right neck dissection + bilateral paratracheal dissection	(‐)	(‐)	- Recurrent cervical mass after 8 months - Mortality by airway suffocation
					- TTF-1, thyroglobulin, CK-19, HBME-1, Galectin-3, BRAF (+) - Ki-67 5% - TERT mutation (+)	- TTF-1, thyroglobulin (+) - Ki-67 3% - NRAS (+)							
Song et al^[25]^	F	49	Palpable cervical mass	(‐)	Right lobe pT1bN1	Left lobe pT1aN1	Lymph node metastasis (PTC/FTC)	(‐)	N/A	TT + CND	(‐)	(+)	No recurrence
					(‐)	CyclinD1, CD-56, thyroglobulin (+), Ki-67 2%							

CND = central lymph node dissection, ETE = extrathyroidal extension, FNA = fine-needle aspiration, FTC = follicular thyroid cancer, HBME-1 = Hector Battifora Mesothelial-1, LL = left lobectomy, N/A = not available, PTC = papillary thyroid cancer, RAI = radioactive iodine therapy, RL = right lobectomy, TERT = telomerase reverse transcriptase, TFT = thyroid function test, TSH = thyroid-stimulating hormone, TT = total thyroidectomy, WNL = within normal limit.

Ye et al retrospectively analyzed the clinicopathological characteristics of synchronous PTC and FTC cohorts at a single center.^[26]^ They reported a higher probability of lymph node metastasis and simultaneous Hashimoto thyroiditis in the PTC-FTC cohort than that in patients with PTC alone. Moreover, the disease-free survival of patients with PTC–FTC was shorter than that of patients with FTC, mainly due to PTC recurrence, although FTC contributed to a higher T stage among patients with PTC–FTC.

It is difficult to diagnose collision tumors during preoperative workup and surgical planning. None of the previous case reports, including ours, could diagnose collision tumors before surgery; they were found incidentally in the pathology reports. Presumably, meticulous pathological examination of the entire specimen is the only method to diagnose collision tumors properly. In our case, PTC was incidentally discovered in the multinodular thyroid. Diagnosing a collision tumor would have been challenging with only hemithyroidectomy, particularly in patients with collision tumors located bilaterally. Notably, previous case reports of PTC and FTC collision tumors have described 3 patients who underwent completion total thyroidectomy after lobectomy.^[3,5,17]^ These findings underscore the importance of meticulous surgical planning in patients with multinodular thyroids. In addition, in cases where a total thyroidectomy is not performed, close postoperative surveillance is essential.

Consensus on managing thyroid collision tumors is lacking owing to the paucity of cases. Some authors have pointed out that collision tumors are more aggressive than individual tumors, even if both are differentiated TCs.^[11,26,27]^ Some authors claim that the treatment of collision tumors should focus on the more aggressive type.^[10]^ In contrast, others insist that each component of a collision tumor should be treated as an independent primary cancer.^[28]^ Most authors agree that treatment should be individualized for patients with collision tumors. Additional case reports and consensus guidelines are needed to establish the proper treatment approach for thyroid collision tumors. Moreover, the prognosis should be assessed cautiously in these patients because PTC and FTC collision tumors have the potential for both lymphatic and hematogenous metastasis.

## 4. Conclusion

This study describes the case of a 65-year-old man who was diagnosed with a collision tumor of the thyroid consisting of papillary and follicular carcinomas on final pathology after total thyroidectomy. Collision tumors of the thyroid, especially synchronous papillary and follicular thyroid carcinomas, are extremely rare and present significant diagnostic and therapeutic challenges. Preoperative diagnosis is difficult; most cases are discovered incidentally on final pathology. Given the distinct biological behavior of each tumor component, individualized surgical planning and meticulous follow-up are imperative. Further accumulation of cases and long-term follow-up data is necessary to establish optimal management strategies for thyroid collision tumors.

## Acknowledgments

We would like to thank Editage (http://www.editage.co.kr) for English language editing.

## Author contributions

**Conceptualization:** Sung-Mo Hur.

**Data curation:** Susie Chin.

**Formal analysis:** Dabin Kim.

**Methodology:** Zisun Kim.

**Resources:** Susie Chin.

**Supervision:** Cheol-Wan Lim.

**Validation:** Cheol-Wan Lim.

**Visualization:** Sung-Mo Hur.

**Writing – original draft:** Dabin Kim.

**Writing – review & editing:** Sung-Mo Hur.
